# Railway Embankments as New Habitat for Pollinators in an Agricultural Landscape

**DOI:** 10.1371/journal.pone.0101297

**Published:** 2014-07-23

**Authors:** Dawid Moroń, Piotr Skórka, Magdalena Lenda, Elżbieta Rożej-Pabijan, Marta Wantuch, Joanna Kajzer-Bonk, Waldemar Celary, Łukasz Emil Mielczarek, Piotr Tryjanowski

**Affiliations:** 1 Institute of Systematics and Evolution of Animals, Polish Academy of Sciences, Kraków, Poland; 2 Institute of Zoology, Poznań University of Life Sciences, Poznań, Poland; 3 Institute of Nature Conservation, Polish Academy of Sciences, Kraków, Poland; 4 Institute of Biology, Pedagogical University of Cracow, Kraków, Poland; 5 Independent researcher, Kraków, Poland; 6 Institute of Environmental Sciences, Jagiellonian University, Kraków, Poland; 7 Institute of Biology, The Jan Kochanowski University, Kielce, Poland; 8 Department of Pomology and Apiculture, Agricultural University in Kraków, Krakow, Poland; University of Marburg, Germany

## Abstract

Pollinating insect populations, essential for maintaining wild plant diversity and agricultural productivity, rely on (semi)natural habitats. An increasing human population is encroaching upon and deteriorating pollinator habitats. Thus the population persistence of pollinating insects and their associated ecosystem services may depend upon on man-made novel habitats; however, their importance for ecosystem services is barely understood. We tested if man-made infrastructure (railway embankments) in an agricultural landscape establishes novel habitats that support large populations of pollinators (bees, butterflies, hoverflies) when compared to typical habitats for these insects, i.e., semi-natural grasslands. We also identified key environmental factors affecting the species richness and abundance of pollinators on embankments. Species richness and abundance of bees and butterflies were higher for railway embankments than for grasslands. The occurrence of bare (non-vegetated) ground on embankments positively affected bee species richness and abundance, but negatively affected butterfly populations. Species richness and abundance of butterflies positively depended on species richness of native plants on embankments, whereas bee species richness was positively affected by species richness of non-native flowering plants. The density of shrubs on embankments negatively affected the number of bee species and their abundance. Bee and hoverfly species richness were positively related to wood cover in a landscape surrounding embankments. This is the first study showing that railway embankments constitute valuable habitat for the conservation of pollinators in farmland. Specific conservation strategies involving embankments should focus on preventing habitat deterioration due to encroachment of dense shrubs and maintaining grassland vegetation with patches of bare ground.

## Introduction

Pollinators play key roles in the ecosystem services essential for maintaining wild plant diversity [1] and agricultural productivity [2]. In the temperate zone the main pollinator groups are bees (Apidae), butterflies (Lepidoptera) and hoverflies (Syrphidae) [3]. Many plant species directly dependent on insect pollination for fruit and seed production [4] may experience pollination limitation if pollinator species are scarce [5]. Therefore, evidence of declines of some native pollinator populations reported throughout Europe and North America [1] are of wide environmental and economical concern. The main factor causing declines of pollinator diversity and abundance is intensification of agriculture [6]. In farmland, the decrease of pollinators' food base and nesting resources are triggered by habitat loss [6]. However, also the cessation of management practices may negatively affects resources needed by pollinators via natural succession (encroachment of shrubs and trees; [7]) and invasion of non-native plants [8].

Great effort has been applied to the development of protection plans in order to sustain the current level of ecosystem services provided by pollinators [9]. Interventions in agriculture, i.e. agri-environmental schemes or the creation of nature reserves in semi-natural habitats, have been devised in the hope that many pollinator populations will survive [10]. However, this approach towards the conservation of species diversity faces many practical problems [11]. Agri-environmental schemes generally benefit pollinators, but their effectiveness depends on where they are implemented [12], what genus or order of pollinators is being targeted [12] or landscape structure [13]. Reserves are frequently located in areas of marginal value for agricultural production, and thus usually play a minor role as a source of pollinator species for farming. Both the creation of reserves and agri-environmental schemes are costly and hence may be limited to the local scale.

A supplementary or alternative solution for the above-mentioned methods is to take advantage of the unrecognized benefits of man-made habitats for pollinator diversity and abundance [14]. Such novel habitats, usually associated with industrial or infrastructural development, may have high conservation value. For example, it has been shown that limestone quarries [15], road verges [16], former open-surface coal mines [17], landfills [18], sandpits [19], gravel-pits [20], gardens [21] or urban parks [22] may be refuges for pollinator populations. Thus, habitats created by human activity may significantly mitigate some of the negative results of industry and agriculture [23].

In the European Union as well as in the United States the overall length of railway lines amounts to more than 200 000 km [24], [25] and is thus a common feature of the landscape. Moreover, EU members have obligated themselves to develop and promote the railway industry [26]. Accordingly, national program of fast speed rail has been launched in the USA [27]. Linear elements in the landscape such as railway lines may play an important role for the functioning of biodiversity and ecosystem services. Linear elements may also act as dispersal corridors [28], reproductive habitats for many organisms [29] but also sink habitats [30]. Although rail lines are frequent elements of many landscapes in the EU and the USA, their contribution to the functioning of biodiversity is not well studied. It is already recognized that embankments are covered by many flowering plant species [31]. The latter suggests that railway embankments may constitute good habitat for many insect species, including pollinators. Moreover, the specific structure of most railways, i.e. a steep embankment with a dry, insolated area at the top and a wetter area at the bottom, creates a strong environmental gradient that may favor different species and therefore increases overall biodiversity [32]. However, to our knowledge, the value of embankments for pollinators has not been studied. Therefore, we explored the value of this habitat for major groups of pollinators by comparing richness and abundance of species with those found in typical pollinator habitat in agricultural landscapes: extensively managed or recently abandoned meadows [33]. We expected that if railway embankments are important then species richness and abundance of pollinators would be similar or higher on embankments than on grasslands. Because railway embankments possess specific features, we also expected that they would be inhabited by different pollinator species than grasslands, adding to overall biodiversity. Further, we identified environmental factors affecting the richness, abundance and species composition of pollinator species on railway embankments to provide recommendations helpful in the management of this habitat for these insects.

## Materials and Methods

### Ethic statement

Permission to access private lands, on which some of the sites were located, was obtained from landowners. Species surveys were conducted according to Polish law.

### Study area

**Figure 1:**
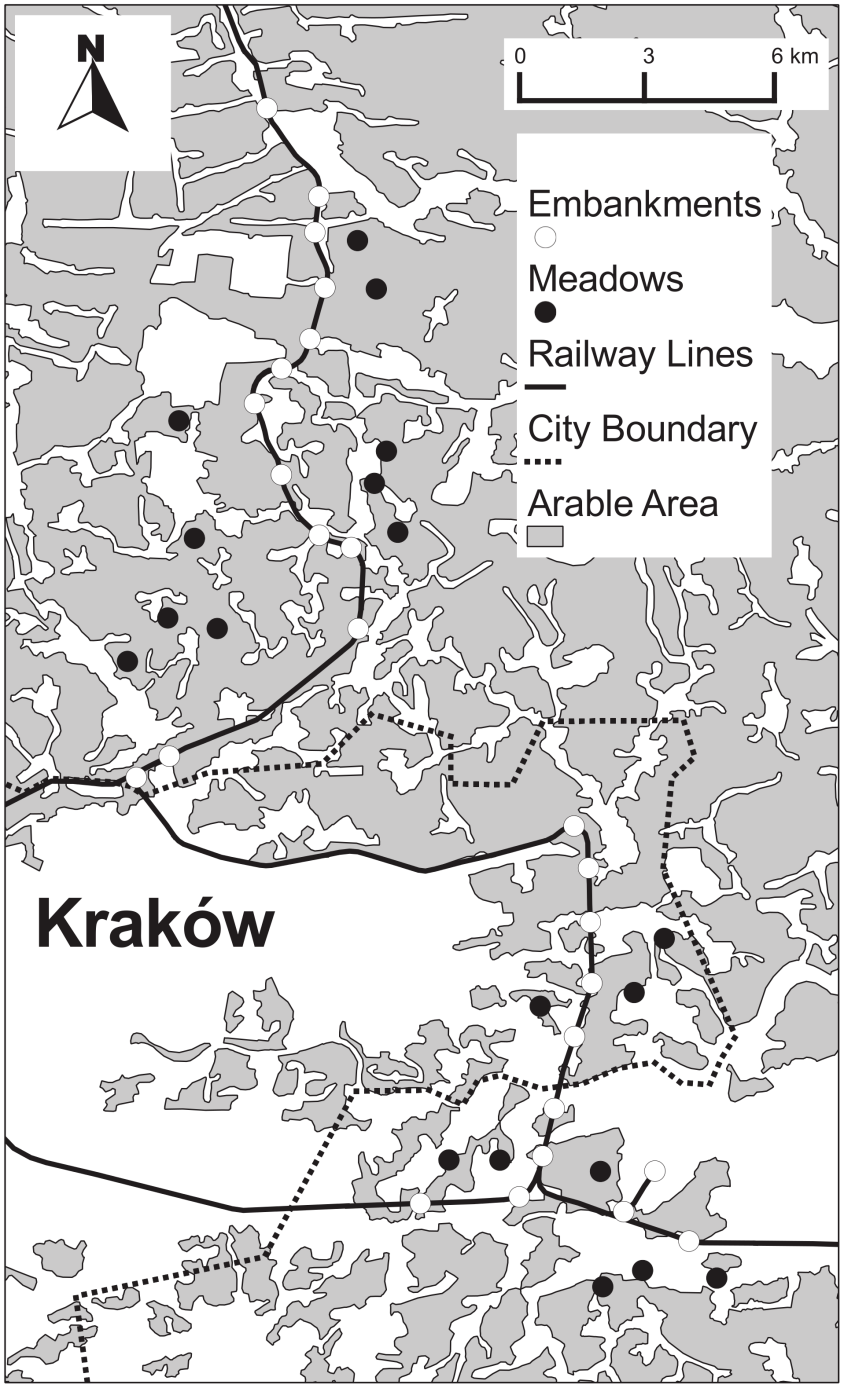
Map indicating the location of the study sites in the Kraków region, SE Poland.

The study was conducted along railway lines in the agricultural region of Kraków, Poland (Fig. 1). All the lines are well-established and were built more than 50 years ago. Using satellite maps we located embankment strips of at least 250 m length. Then, we randomly selected 25 of these embankments (Fig. 1). All selected embankments were separated by a mean distance of 1330±467 m (mean ± SE, range: 779–2359 m).

### Pollinator surveys

A 200 m transect was established at each strip for pollinator surveys [34]. In total, there were 25 transects along the embankments (Fig. 1). Each transect was located in the middle of a given strip. Bees and hoverflies were swept on each transect in May, at the turn of June and July and in August. During three transect walks on each site, the collectors walked at a slow pace making 500 sweeps to standardize sweeping effort. Sweeps encompassed all flowering plants at transects. Individuals were sorted, pinned and dried, prior to species identification, except for species protected by law. Butterflies were also counted on each transect from May to August on three occasions. Pollinators were surveyed during clear, warm and calm weather conditions on all sampling days. The order in which the transects were sampled was random. Each transect was visited during different parts of the day throughout the season.

To compare the number of pollinator species and their abundance on embankments with those inhabiting grasslands we established a further 19 transects on extensively managed grassland or recently abandoned grassland (<5 years; Fig. 1). We chose grasslands in the vicinity of the embankments (mean ± SE distance to the nearest railway lines was 1511±647 m, range: 816–2947 m) to keep such factors as bedrock, climate and landscape composition similar to the railway transects. We chose extensively managed grassland and recently abandoned grassland because earlier studies showed that they are one of the most important and widespread habitats for pollinators [7]. Agriculture in southern Poland is structured so that fields and grasslands are usually small and elongated and thus somewhat similar to railway embankments at least in shape (the mean ± SE ratio of length to width of grassland was 4.88±3.55).

### Environmental variables measured for embankment

The following environmental variables potentially affecting pollinators' food base and nesting resources were determined for embankments: bare ground cover, grassland cover, human settlement cover, species richness of native flowering plants, species richness of non-native flowering plants, length of railway lines, angle of slopes, length of slopes, shrub cover, vegetation height, water reservoir cover and woodland cover (Table 1). Bare ground and shrub cover were estimated as percentages (0–100%) of embankment area. Grassland, human settlement, water reservoir and woodland covers were measured as percentages (0–100%) in a 200 m buffer around the transects. Length of railway lines were measured as truck length (m) per 1 m^2^ of the buffer. Variables measured in the buffer were read from aerial photos digitalized in Quantum GIS software and supported by direct measurements in the field by GPS. Angle (rad) and length (m) were measured in the middle and at both ends of transects and then the mean was used in analyses. Plant species richness and plant height (cm) were measured in six circular plots of 1 m diameter (0.79 m^2^) established at each transect with a distance of 40 m between the plots. Because number of plant species and plant cover were highly, positively correlated (as indicated our preliminary study), thus to avoid multicollinearity problems, we decided to noticed only plant species richness. Plant surveys were done twice during the study in May and in July.

**Table 1:** Independent variables measured on embankment sites.

Independent variables	Mean± SD (min. - max.)
bare ground cover (%)	3.72±7.32 (0–32)
grassland cover (%)	35.56±20.05 (9.52–79.49)
human settlement cover (%)	15.95±11.61 (0.03–42.42)
species richness of native flowering plants (no. species)	21.32±6.46 (6–32)
species richness of non-native flowering plants (no. species)	1.40±0.90 (0–4)
length of railway lines (10^−3^ m/m^2^)	2.14±1.38 (0.83–6.21)
angle of slopes (rad)	0.59±0.15 (0.20–0.82)
length of slopes (m)	8.59±3.34 (2.37–18.00)
shrub cover (%)	14.72±20.19 (0–70)
vegetation height (cm)	51.80±8.52 (40–70)
water reservoir cover (%)	0.96±3.23 (0.0–14.6)
woodland cover (%)	2.22±4.95 (0.00–18.33)

Mean ± standard deviation (SD) with minimum and maximum values are shown.

### Analysis

We square-rooted dependent variables in order to normalize distributions, to linearize relationships and to reduce the effects of outlying observations [35]. Independent variables were standardized (mean of zero and a standard deviation of one) to allow for direct comparison of function slopes between them [35]. Our primary analysis showed that vegetation height and length of slopes as well as grassland cover and shrub cover were correlated (*r_S_* = −0.644 and *r_s_* = 0.657 respectively; we used criterion of correlations *r_S_*>|0.600|). To avoid multicollinearity we expressed vegetation height and grassland cover as residuals. Before analysis we checked if there was any spatial autocorrelation in the data by calculating Moran's statistics on correlograms (Fig. S1,S2; [36]). However, we did not find evidence for statistically significant autocorrelation thus we used traditional statistics.

To identify factors affecting pollinator richness and abundance on embankments we used a model selection procedure based on information theory [37]. The Akaike information criterion corrected for small sample size (AICc) was used to identify the most parsimonious models from each candidate set. Then, we ranked all models according to their ΔAICc values and used those with the lowest AICc together with associated weight values (probability that a given model is the best) as the best model describing the data. We considered models with ΔAICc lower than two as equally good [37]. We used model averaging for estimates of function slopes of parameters of interest [37]. Finally, the model weights were used to define the relative importance of each explanatory variable across the full set of models evaluated by summing weight values of all models that include the explanatory variable of interest [37]. We considered that the function slopes (betas) were significant if their 95% confidence intervals (95% CI) did not overlap with zero. Statistical models were built separately for each pollinator group (bees, butterflies and hoverflies; 4095 models tested for each pollinator group). Procedures of model selection and averaging according to the AICc were run in SAM 4.0 statistical software [38].

We used the Wilcoxon rank sum test to compare the number of pollinator species and their abundances among embankments and reference grasslands as well as the abundance of particular species in these two habitats. We applied χ^2^ tests or the Fisher exact test (when frequencies were lower than ten) to examine the proportion of transects at which a given species was recorded for embankments and grasslands. We used the redundancy analysis (RDA) to find how species composition was related to the habitat type (embankments and reference grasslands) as well as to environmental variables measured on embankments. We applied the RDA analysis with forward selection of variables on the basis of their permutational p-values and on AIC criterion. The Wilcoxon test, χ^2^ tests, Fisher test and RDA analysis were conducted separately for pollinator groups in R software [39].

## Results

### Comparison of embankments and grasslands

**Figure 2:**
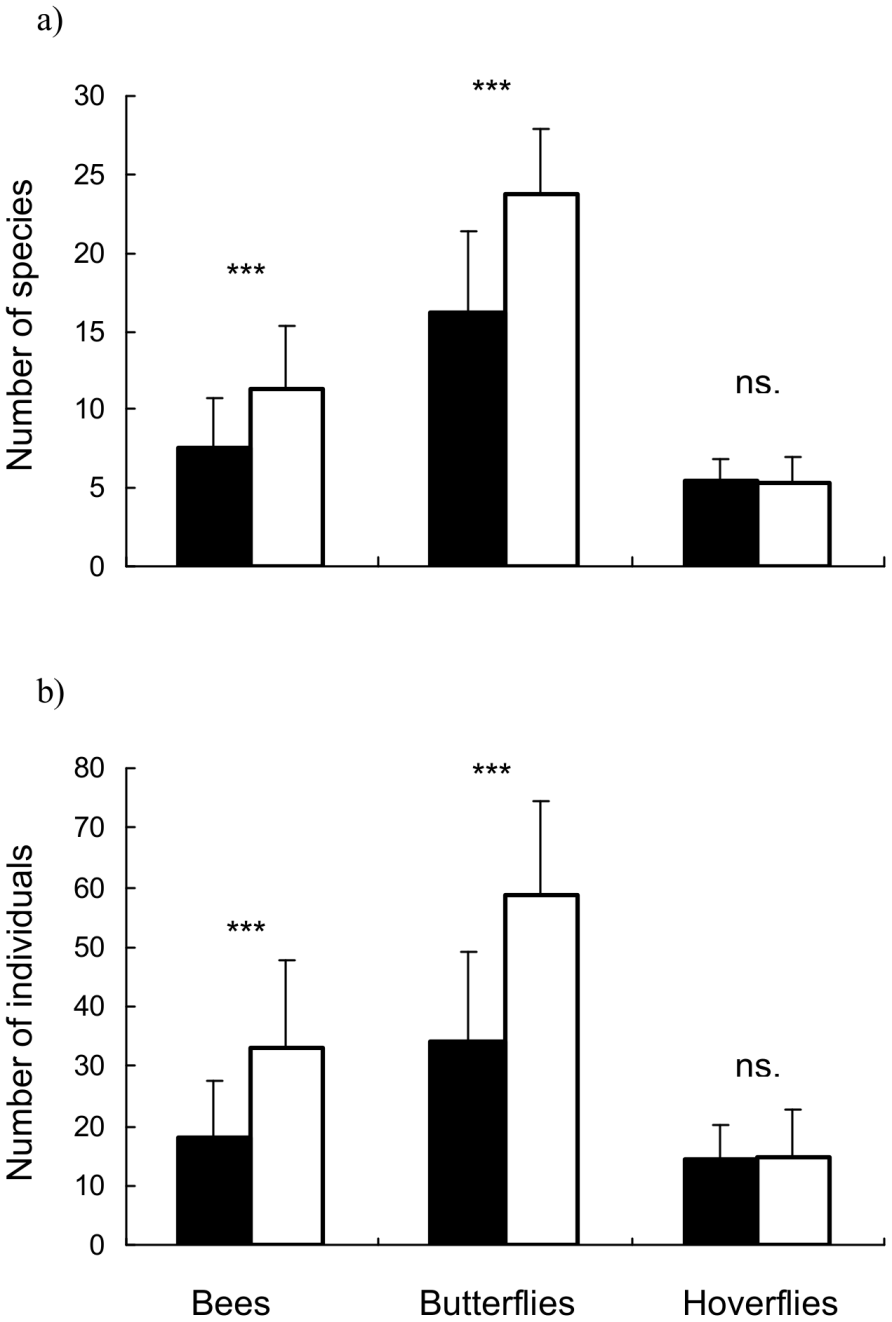
Mean (±SD) pollinator species richness (a) and abundance (b) on railway embankments (closed bars) and grasslands (open bars). ***P<0.001.

Bee and butterfly species richness were higher for about 30% in embankment transects than grassland transects (bees: W =   = 98.5, p<0.001; butterflies: W = 57, p<0.001; Fig 2a), however hoverfly species richness did not differ between habitats (W = 246, p = 0.845; Fig. 2a). The same patterns were found for abundance of pollinator groups, there were about 40% more bee and butterfly individuals in embankments than grassland transects (bees: W = 92.5, p<0.001; butterflies: W = 53, p<0.001; hoverflies: W = 248, p = 0.812; Fig. 2b). Redundancy analysis showed that habitat type (embankments vs. grasslands) significantly explained 3.4% of bee (F_1,42_ = 1.47, p = 0.01) and 7.3% of butterflies (F_1,42_ = 3.31, p = 0.005) variation in species composition. The difference in composition of species was not significant for hoverflies (F_1,42_ = 1.18, p = 0.115). Altogether, 46 bee (out of 100), 13 butterfly (out of 66) and 20 hoverfly (out of 46) species were unique for embankments (Table S1). A total of 18, 4 and 10 species, respectively, were unique for grasslands (Table S1). However, these differences were statistically non-significant (bees: χ^2^ = 2.62, p = 0.105; butterflies: F. exact, p = 0.239; hoverflies: χ^2^ = 0.73, p = 0.394). There were 36 species of bees, 49 species of butterfly and 16 species of hoverfly that occurred on both embankments and grasslands (Table S1). Among these 16 were significantly more abundant and/or had a higher incidence on railway embankments (3 bee, 12 butterfly and 1 hoverfly species; Table S1). Also, there was one bee species that occurred significantly more often on grassland transects (Table S1). The remaining pollinators (84 species) did not differ significantly in density or occurrence for embankment vs. grassland transects (Table S1).

### Embankment characteristics affecting pollinators

The model selection based on Akaike's criterion showed that one model explained bee species richness on embankments (Table 2,3). The model explained 72% of variation in bee species richness. Explanatory variables that were present in the model included species richness of non-native flowering plants, bare ground cover, shrub cover and woodland cover. Model selection identified twelve equally good models describing the abundance of bees on embankments (Table 2,3). These best models explained 66% of variation on average. Explanatory variables present in all best models included shrub cover and bare ground cover. The abundance of bees was also dependent on species richness of native flowering plants, grassland cover, length of slope, vegetation height, woodland cover, species richness of non-native flowering plants, length of railway lines and human settlement cover (Table 2,3). The RDA analysis showed that species richness of non-native flowering plants and woodland cover explained 5.16% of the variance in composition of bee populations (F_2,22_ = 1.65, p = 0.01).

**Table 2:** Best models describing bee, butterfly and hoverfly species richness and abundance by variables on railway embankments.

No.	Model	k	r^2^	AICc			Δ AICc	w
	*Bee species richness*							
1	ground (↑) + nna (↑) + shrub (↓) + wood (↑)	4	0.72	29.55			0.00	0.191
	*Bee abundance*							
1	ground (↑) + na (↓) + shrub (↓)	3	0.64	70.34			0.00	0.037
2	grass (↓) + ground (↑) + shrubs (↓)	3	0.64	70.49			0.15	0.034
3	ground (↑) + lslop (↑) + nna (↑) + shrub (↓)	4	0.69	70.54			0.20	0.033
4	ground (↑) + shrub (↓)	2	0.59	70.55			0.20	0.033
5	grass (↓) + ground (↑) + na (↓) + shrub (↓)	4	0.68	71.24			0.9	0.022
6	ground (↑) + height (↑) + shrub (↓)	3	0.62	71.50			1.16	0.021
7	ground (↑) + lslop (↑) + nna (↑) + shrub (↓) + wood (↓)	5	0.72	71.55			1.21	0.020
8	ground (↑) + nna (↑) + shrub (↓) + wood (↓)	4	0.67	71.63			1.29	0.019
9	ground (↑) + set (↓) + shrub (↓)	3	0.62	71.80			1.46	0.018
10	grass (↓) + ground (↑) + rail (↑) + shrubs (↓)	4	0.67	71.84			1.50	0.017
11	grass(↓) + ground(↑) + lslop(↑) + nna(↑) + shrub(↓)	5	0.72	71.99			1.65	0.016
12	ground (↑) + shrub (↓) + wood (↓)	3	0.62	72.13			1.79	0.015
	*Butterfly species richness*							
1	ground (↓)+ na (↑)	2	0.60	12.90		0.00		0.083
2	ground (↓)+ na (↑) + wood (↓)	3	0.63	14.10		0.19		0.046
	*Butterfly abundance*							
1	na (↑)	1	0.35	68.23		0.00		0.039
2	aslope (↑) + na (↑)	2	0.42	68.29		0.06		0.038
3	ground (↓) + na (↑)	2	0.42	68.58		0.34		0.033
4	aslop (↑) + ground (↓) + na (↑)	3	0.48	69.05		0.82		0.026
5	na (↑) + nna (↓)	2	0.40	69.40		1.17		0.022
6	lslop (↑) + na (↑)	2	0.38	70.17		1.93		0.015
7	na (↑) + wa (↓)	2	0.38	70.20		1.97		0.015
8	aslop (↑) + na (↑) + wa (↓)	3	0.45	70.22		1.99		0.014
	*Hoverfly species richness*							
1	wood (↑)	1	0.21	19.66			0.00	0.031
2	aslop (↓) + wood (↑)	2	0.29	20.10			0.44	0.025
3	lslope (↓) + wood (↑)	2	0.27	20.79			1.13	0.017
4	grass (↑) + wood (↑)	2	0.27	20.80			1.14	0.017
5	ground (↑) + wood (↑)	2	0.25	21.30			1.64	0.014
6	ground (↑) + grass (↑) + wood (↑)	3	0.34	21.31			1.65	0.013
7	na (↑) + wood (↑)	2	0.24	21.63			1.98	0.011
	*Hoverfly abundance*							
1	lslope (↓)	1	0.12	73.26			0.00	0.048
2	aslope (↓)	1	0.04	75.24			1.98	0.018

For each model the number of predictors (k), variance explained by the model (r^2^), the Akaike information criterion score (AICc), the difference between the given model and the most parsimonious model (Δ) and Akaike weight (w) are listed. Explanations of variable codes: bare ground cover – ground, grassland cover – grass, human settlement cover – set, species richness of native flowering plants – na, species richness of non-native flowering plants – nna, length of railway lines – rail, angle of slopes – aslop, length of slopes – lslop, shrub cover – shrub, vegetation height – height, water reservoir cover – wa, woodland cover – wood. (↑) – positive, significant relationship; (↓) – negative, significant relationship.

**Table 3:** Estimates of the function slopes of variables present in the most parsimonious models describing bee, butterfly and hoverfly species richness and abundance by variables on railway embankments.

Variable	Importance	Estimate	SE	Lower 95% LC	Upper 95% CL
*Bee species richness*					
nna	0.979	0.301	0.087	0.131	0.472
ground	0.977	0.269	0.079	0.113	0.424
shrub	0.957	−0.268	0.084	−0.432	−0.104
wood	0.705	0.18	0.057	0.068	0.292
*Bee abundance*					
shrub	0.998	−0.879	0.218	−1.306	−0.453
ground	0.970	0.627	0.190	0.254	1.000
na	0.444	−0.381	0.101	−0.579	−0.183
grass	0.362	−0.289	0.070	−0.426	−0.153
lslop	0.304	0.243	0.055	0.134	0.351
wood	0.250	−0.214	0.046	−0.304	−0.125
height	0.203	0.183	0.041	0.104	0.263
nna	0.192	0.181	0.039	0.106	0.257
rail	0.185	0.166	0.037	0.095	0.238
set	0.180	−0.168	0.036	−0.240	−0.097
*Butterfly species richness*					
na	1.000	0.329	0.069	0.194	0.464
ground	0.761	−0.141	0.047	−0.233	−0.049
wood	0.331	−0.082	0.020	−0.121	−0.043
*Butterfly abundance*					
na	0.980	0.674	0.193	0.295	1.053
aslop	0.403	0.286	0.076	0.137	0.436
ground	0.355	−0.257	0.065	−0.385	−0.129
nna	0.229	−0.180	0.044	−0.266	−0.095
water	0.209	−0.153	0.038	−0.227	−0.078
lslop	0.197	0.148	0.038	0.074	0.223
*Hoverfly species richness*					
wood	0.756	0.444	0.053	0.056	0.262
grass	0.345	0.101	0.026	0.050	0.151
lslop	0.311	−0.087	0.022	−0.130	−0.045
aslop	0.308	−0.090	0.022	−0.133	−0.046
ground	0.292	0.087	0.021	0.045	0.129
na	0.213	0.063	0.017	0.030	0.097
*Hoverfly abundance*					
lslop	0.471	−0.326	0.096	−0.514	−0.137
aslop	0.279	−0.226	0.062	−0.347	−0.105

Standard errors (SE) and 95% confidence limits (CL) are also presented. Name of variables as in Tab. 2.

Model selection identified two models describing butterfly species richness on embankments (Table 2,3). The models explained 62% of variation on average. Explanatory variables present in these models included the species richness of native flowering plants and bare ground cover. The abundance of butterflies was also dependent on wood cover. Model selection identified eight equally good models describing the abundance of butterflies on embankments (Table 2,3). These best models explained 41% of variation on average. Species richness of native flowering plants was present in all best models. The abundance of butterflies was also dependent on angle of slope, bare ground cover, species richness of non-native flowering plants, water cover in a landscape and length of slope. The RDA analysis revealed that butterfly species composition on embankments were significantly dependent on species richness of native flowering plants (F_1,23_ = 1.54, p = 0.02) which explained 2.18% of the variance in butterfly composition.

Model selection identified seven models describing hoverfly species richness on embankments (Table 2,3). These best models explained 27% of variation on average. Explanatory variable present in the models was woodland cover. The species richness of hoverflies was also dependent on grassland cover, length of slopes, angle of slopes, bare ground cover and species richness of native flowering plants. Model selection identified two equally good models describing the abundance of hoverflies (Table 2,3). These best models explained 8% of the variation on average. The abundance of hoverflies was dependent on length of slope and angle of slope. The RDA analysis showed that angle of slopes, railway lines and woodland cover explained 13.3% of the variance in composition of hoverfly populations (F_3,21_ = 2.23, p = 0.005).

## Discussion

### Embankments vs. grasslands

Railway embankments are linear habitats that are typically built of crushed stone or different sized gravel, constructed in a way that leads to drier, warmer condition at the top of the embankment whereas the bottom is colder and wetter [40]. Moreover, changes over time, e.g. succession, and constant disturbance during maintenance often adds to the substantial habitat mosaic (D. Moroń, personal observations). Thus, railway embankments can be a significant habitat for many species, especially in highly modified landscapes. Our results demonstrated that railway embankments are important habitats for pollinators in an agricultural landscape. The total number of bee and butterfly species and their abundances were higher for embankments than grasslands. However, pollinator communities on railway embankments were fairly similar to grassland communities. The abundance and incidence of some pollinators was higher on embankments than on grasslands (17% of shared species). Thus, railway embankments may be an important habitat for some key pollinators such as *Bombus lapidarius* and *B. terrestris* (Table S1). Accordingly, the expectation that flowering crops, e.g. alfalfa or oilseed rape, will receive more pollination services from pollinators in landscapes with railway embankments should be tested in future studies.

### Embankment characteristics affecting pollinators

Environmental properties of embankments significantly influenced the richness of pollinator species as well as their abundance. However, the factors explained little variation in pollinator species composition on embankments (7% on average). The low explained variance indicate that abundance of most pollinator species similarly responded to the environmental variables. There are two groups of limiting factors for insect pollinator populations, i.e. those related to nesting requirements (for bees) and those related to foraging requirements (for bees, butterflies and hoverflies; [41]). Here we found that bare ground cover positively affected bee richness and abundance on embankments. Because most bee species are ground-nesters (about 95% of bee species; [42]) such microhabitats significantly contribute to the overall pool of bee species [41]. Disturbances caused by the maintenance or repair of embankments can create patches of bare ground, exceeding even 30 percent of the area (D. Moroń, personal observation). However, this factor has an opposite effect on butterfly species richness and abundance. Butterflies, contrary to bees, do not usually rely on exposed ground but rather on nectar sources or larval host plants growing on embankments. Bees as well as butterflies are dependent on flowering plants as sources of pollen and nectar [1], however only butterfly populations positively depended on species richness of native flowering plants on embankments. It is difficult to establish if this is because of close relationships of plants and pollinators or because butterflies and plants tend to respond to the same environmental factors [43]. Interestingly, bee species richness was positively affected by species richness of non-native flowering plants. This result seems to contradict earlier studies showing a strong, negative impact of invasive plants on pollinators (e.g. [8]). However, invasive species rarely create dense mono-specific stands on embankments (D. Moroń, personal observation). Frequent disturbance of above-ground as well as below-ground biota, observed on embankments, may weaken the competitive abilities of invasive species [44]. This result suggests that the effect of invasive alien species on pollinators may not be linear. When the cover of invasive plants is low it might positively affect pollinator populations by providing more diverse resources available at different times of the year but if the cover of invasive species increases the native flowering plants become excluded and thus populations of pollinators decline. Shrubs negatively affected the number of bee species and their abundance. Dense shrubs could diminish the suitability of embankments for, e.g. pioneer or specialist bee species by changing microclimatic conditions and plant species composition. Shrubs may also mediate higher predation rate by birds that hunt pollinators and their larvae and that use shrubs as nest or perching sites [45]. Thus, it is possible that predation rate may also be responsible for the lower number of individuals of bees on embankments covered by shrubs. Landscape-scale variables were absent among the most important factors influencing pollinator populations on railway embankments. Many pollinators are small-bodied species with a very limited dispersal ability [46] thus local factors may to be more influential than the landscape surrounding embankments. [28]. In our study the richness of bee and hoverfly species were positively dependent on woodland cover. Many hoverflies occurring on embankments prefer open, sunny areas. However, increasing woodland area may boost species richness of this group because a large number of hoverfly species prefer woodland habitats, i.e. larvae need decaying trunks or rot-holes to complete their life cycle [47]. Also, females of some bees (∼5% of species; [42]) use different kinds of pre-existing cavities in wood to construct their nests. Because nesting resources are one of the most limiting factor for bee population, thus increasing woodland cover near the embankments may enrich the community.

### Conclusions

As we have shown, some infrastructure development may also bring positive effects on local biodiversity by the creation of novel habitats. Thus, it is worth putting more emphasis on finding the positive effects of human activity and working out solutions which may make this activity beneficial for wild animals and plants [48]. For example, it would be beneficial for pollinators if embankments were managed in order to avoid habitat deterioration by dense shrub growth which seems essential especially for bees. Also, the maintenance of a mosaic patches of exposed ground as well as woodland vegetation in the landscape is recommended. Fortunately, shrubs are removed during the regular maintenance of railways, while embankment repair frequently results in creating exposed ground or sparse vegetation (D. Moroń, personal observation). This indicates that railway embankment habitat is unintentionally managed and disturbed in a manner favorable for different species. However, abandoned railway lines (over 2000 km during 10 years in Poland; [24]) require additional management effort to sustain their value for pollinators. Because species richness of flowering plants increases pollinator species number and abundance on embankments, we recommend sowing seed mixtures of wild flowers [49]. Despite that the mean number of invasive species was slightly higher on embankments compared to control grasslands (1.4. vs. 0.9, respectively), there was a lack of a negative impact on pollinators. This implies that there is currently no need for laborious and costly eradication of invasive species from embankments. However the exotic species present on embankments may invade habitats located close to railway lines [50] and have a more detrimental effect on pollinators [8]. Somewhat surprisingly, embankment properties such as slope length and angle did not affect most of pollinator groups. Thus, our results indicate that embankments of different shape and size are probably of similar suitability for pollinator conservation.

Having recognized the positive aspects of railways for pollinators, the possible threats for pollinator biodiversity should also be mentioned. Railway traffic can cause pollinator mortality and in this way lower population abundance. Railway transport can also be a serious source of different kinds of pollution [40] which may negatively impact pollinator richness and abundance [51], [52]. Pollution also includes non-selective herbicides used to maintain tracks which, in turn, may negatively impact pollinator populations [53], e.g. by lowering flowering plant richness and abundance. However, our study demonstrated that possible benefits (larger populations of pollinators) brought by the existence of railway embankments probably overcome these negative phenomena.

In summary, the ecosystem functioning of agricultural landscapes may be enhanced (e.g. a higher number of species and their diversity, more pollinated natural vegetation and crops) by the presence of embankments and their proper management. Railway embankments may thus be a text-book example of man-made alterations in the environment that alleviate conflicts between the demands of civilization and conservation of wildlife and biodiversity.

## Supporting Information

Figure S1 **Moran's I correlograms for species richness of bees (a), butterflies (b) and hoverflies (c).** Points represent Moran's I values. Envelopes of 95% confidences intervals are shown in dark-grey, envelopes of maximu Moran's I are shown in light-grey. None of spatial autocorrelations were significant after using Bonferroni correction.
(DOC)

Figure S2 **Moran's I correlograms for abundance of bees (a), butterflies (b) and hoverflies (c).** Points represent Moran's I values. Envelopes of 95% confidences intervals are shown in dark-grey, envelopes of maximum Moran's I are shown in light-grey. None of spatial autocorrelations was significant after using Bonferroni correction.
(DOC)

Table S1 **List of all wild bee (a), butterfly (b) and hoverfly (c) species recorded within transects on embankments and grasslands.** Abundance is the mean number of individuals per transect in which a given species was recorded. Occurrence is the number of transects with a given species. The total number of sites for embankments and grasslands was 25 and 19, respectively. A Wilcoxon rank sum test was used for abundance analysis. Fisher exact (F. exact) tests were used when frequencies were lower than 10, otherwise χ2 were used for occurrence analysis.
(DOC)
