# Supplementary material for: Railway Embankments as New Habitat for Pollinators in an Agricultural Landscape
Source: PLoS One. 2014 Jul 23;9(7):e101297. doi: 10.1371/journal.pone.0101297 (PMC4108474; doi:10.1371/journal.pone.0101297)
Supplement: Figure S1 — Moran's I correlograms for species richness of bees (a), butterflies (b) and hoverflies (c). Points represent Moran's I values. Envelopes of 95% confidences intervals are shown in dark-grey, envelopes of maximu Moran's I are shown in light-grey. None of spatial autocorrelations were significant after using Bonferroni correction. (DOC) [file pone.0101297.s001.doc]

Figure S1. Moran’s *I* correlograms for species richness of bees (a), butterflies (b) and hoverflies (c). Points represent Moran’s *I* values. Envelopes of 95 % confidences intervals are shown in dark-grey, envelopes of maximu Moran’s *I* are shown in light-grey. None of spatial autocorrelations were significant after using Bonferroni correction.

a)


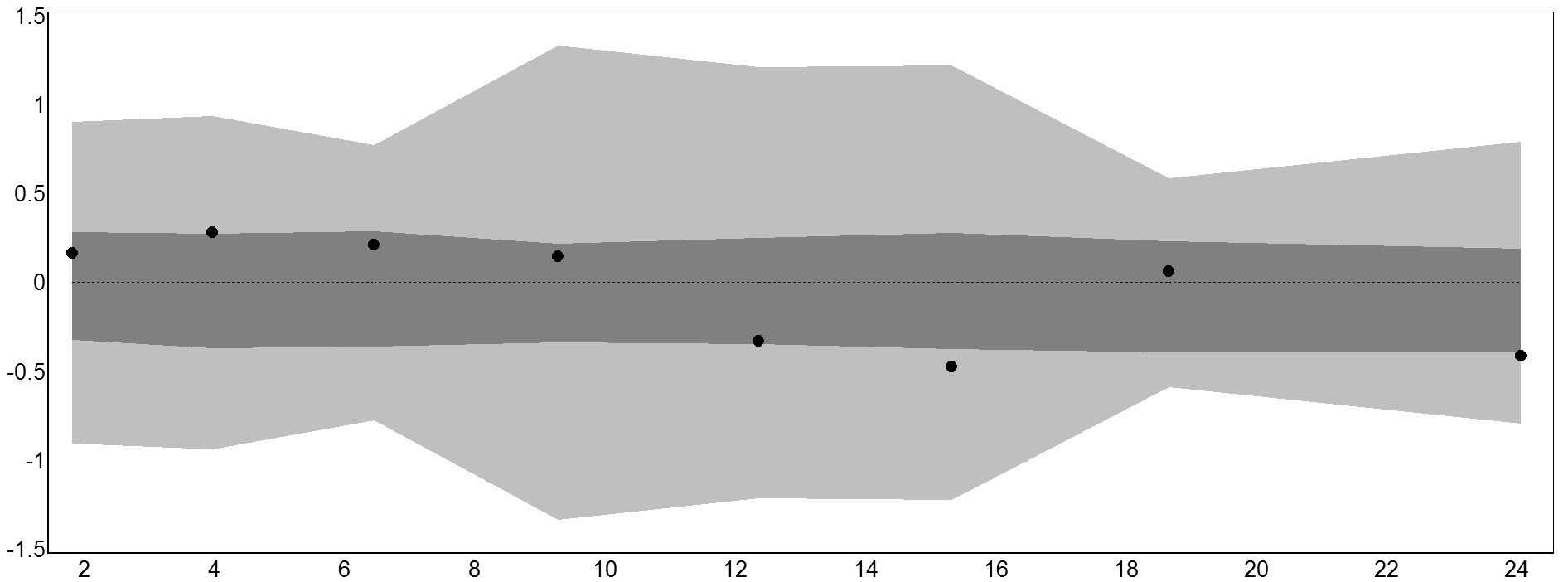


b)

Moran’s *I*

Moran’s *I*


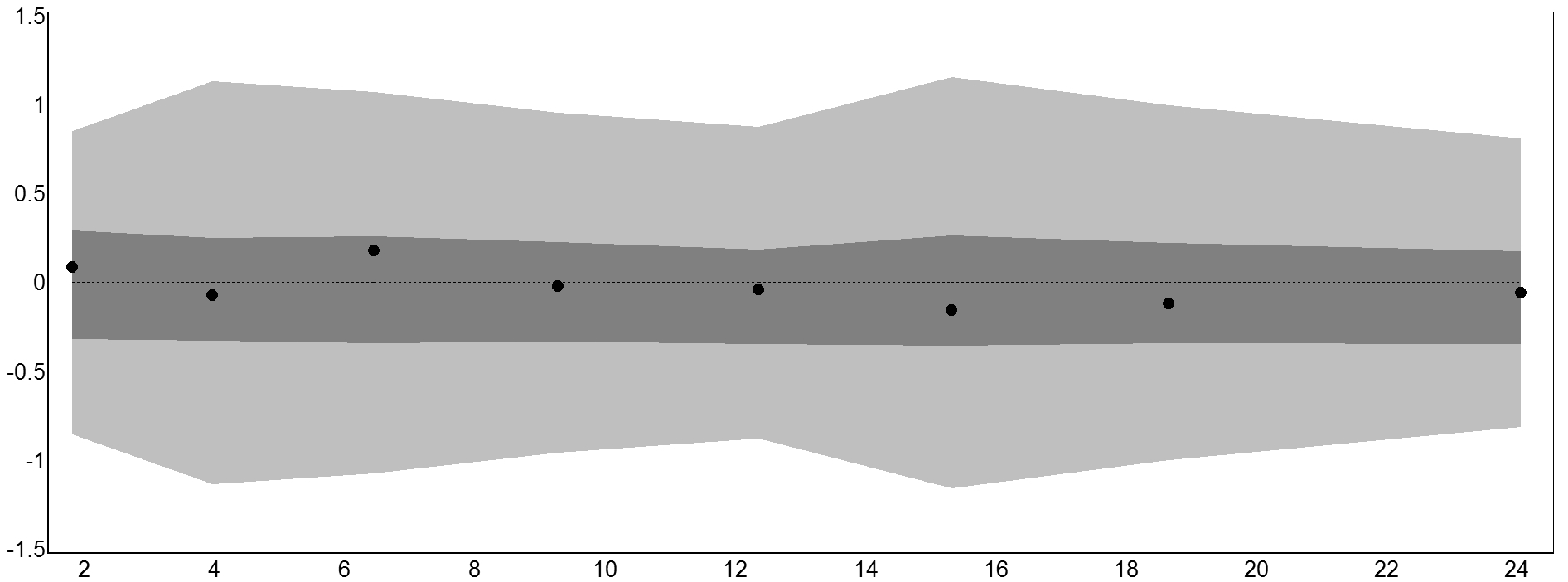


c)


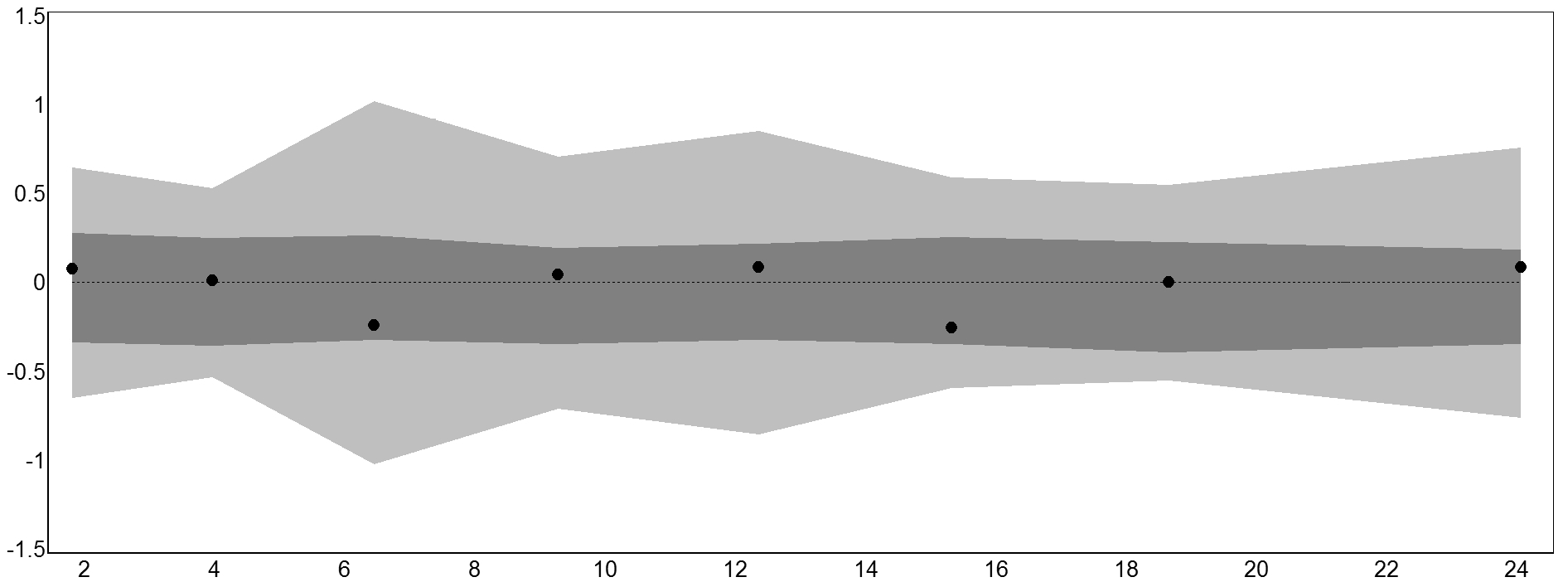


Distance
