# Supplementary material for: Railway Embankments as New Habitat for Pollinators in an Agricultural Landscape
Source: PLoS One. 2014 Jul 23;9(7):e101297. doi: 10.1371/journal.pone.0101297 (PMC4108474; doi:10.1371/journal.pone.0101297)
Supplement: Table S1 — List of all wild bee (a), butterfly (b) and hoverfly (c) species recorded within transects on embankments and grasslands. Abundance is the mean number of individuals per transect in which a given species was recorded. Occurrence is the number of transects with a given species. The total number of sites for embankments and grasslands was 25 and 19, respectively. A Wilcoxon rank sum test was used for abundance analysis. Fisher exact (F. exact) tests were used when frequencies were lower than 10, otherwise χ2 were used for occurrence analysis. (DOC) [file pone.0101297.s003.doc]

Table S1. List of all wild bee (a), butterfly (b) and hoverfly (c) species recorded within transects on embankments and grasslands. Abundance is the mean number of individuals per transect in which a given species was recorded. Occurrence is the number of transects with a given species. The total number of sites for embankments and grasslands was 25 and 19, respectively. A Wilcoxon rank sum test was used for abundance analysis. Fisher exact (F. exact) tests were used when frequencies were lower than 10, otherwise χ^2^ were used for occurrence analysis.

a)

| Species | Abundance | | | | Occurrence | | | |
| --- | --- | --- | --- | --- | --- | --- | --- | --- |
|  | Embankment | Grassland | Statistic | P | Embankment | Grassland | Statistic | P |
| *Andrena agilissima* | 1.0 | - | - | - | 1 | 0 | F. exact | 1.000 |
| *Andrena decipiens* | - | 1.0 | - | - | 0 | 1 | F. exact | 0.444 |
| *Andrena dorsata* | 1.7 ± 1.2 | - | - | - | 3 | 0 | F. exact | 0.262 |
| *Andrena falsifica* | - | 1.0 | - | - | 0 | 1 | F. exact | 0.444 |
| *Andrena flavipes* | 1.7 ± 0.8 | 2.0 ± 1.4 | W = 8 | 0.876 | 2 | 7 | F. exact | 0.076 |
| *Andrena floricola* | - | 1.0 | - | - | 0 | 1 | F. exact | 0.444 |
| *Andrena gelriae* | 2.0 ± 1.7 | 1.0 ± 0.0 | W = 2 | 0.683 | 3 | 2 | F. exact | 1.000 |
| *Andrena gravida* | 2.0 ± 1.4 | - | - | - | 0 | 2 | F. exact | 0.203 |
| *Andrena hattorfiana* | 1.0 | - | - | - | 1 | 0 | F. exact | 1.000 |
| *Andrena humilis* | 1.0 | - | - | - | 1 | 0 | F. exact | 1.000 |
| *Andrena helvola* | 3.0 | - | - | - | 1 | 0 | F. exact | 1.000 |
| *Andrena labialis* | - | 1.0 | - | - | 0 | 1 | F. exact | 0.444 |
| *Andrena labiata* | 1.0 | 1.5 ± 0.7 | W = 1.5 | 1.000 | 1 | 2 | F. exact | 1.000 |
| *Andrena lathyri* | 1.0 | - | - | - | 1 | 0 | F. exact | 1.000 |
| *Andrena minutula* | 1.2 ± 0.4 | 2.0 ± 1.0 | W = 11.5 | 0.228 | 5 | 3 | F. exact | 1.000 |
| *Andrena minutuloides* | 2.3 ± 1.0 | 1.8 ± 1.3 | W = 9.5 | 0.337 | 6 | 5 | F. exact | 1.000 |
| *Andrena niveata* | 1.0 | - | - | - | 1 | 0 | F. exact | 1.000 |
| *Andrena pilipes* | 1.0 ± 0 | 1.0 | - | - | 2 | 1 | F. exact | 1.000 |
| *Andrena pontica* | 3.0 ± 2.0 | - | - | - | 3 | 0 | F. exact | 0.262 |
| *Andrena proxima* | 2.7 ± 2.1 | 3.0 | W = 2 | 1.000 | 3 | 1 | F. exact | 0.631 |
| *Andrena pusilla* | 2.0 | - | - | - | 1 | 0 | F. exact | 1.000 |
| *Andrena subopaca* | 1.0 ± 0.0 | 1.0 ± 0.0 | - | - | 4 | 2 | F. exact | 1.000 |
| *Andrena viridescens* | 2.0 | 1.0 | W = 0 | 1.000 | 1 | 1 | F. exact | 1.000 |
| *Anthophora aestivalis* | 1.0 | 1.0 | - | - | 1 | 1 | F. exact | 1.000 |
| *Bombus hortorum* | 3.0 ± 2.8 | 1.0 | W = 0.5 | 1.000 | 2 | 1 | F. exact | 1.000 |
| *Bombus lapidarius* | 9.3 ± 6.7 | 5.5 ± 4.8 | W = 111.5 | **0.033** | 22 | 17 | $\chi^{2}=0.0$ | 0.970 |
| *Bombus muscorum* | - | 4.0 | - | - | 0 | 1 | F. exact | 0.444 |
| *Bombus pascuorum* | 3.7 ± 2.5 | 1.7 ± 1.2 | W = 12.5 | 0.085 | 10 | 6 | F. exact | 0.773 |
| *Bombus ruderarius* | 2.0 | 1.0 | W = 0 | 1.000 | 1 | 1 | F. exact | 1.000 |
| *Bombus subterraneus* | 3.0 | - | - | - | 1 | 0 | F. exact | 1.000 |
| *Bombus sylvarum* | 2.9 ± 4.1 | 2.2 ± 2.1 | W = 70 | 0.817 | 11 | 12 | $\chi^{2}=0.5$ | 0.483 |
| *Bombus terrestris* | 8.6 ± 5.8 | 5.7 ± 6.3 | W = 90.5 | **0.040** | 22 | 14 | $\chi^{2}=0.2$ | 0.698 |
| *Ceratina cyanea* | 1.0 ± 0.0 | 1.0 | - | - | 2 | 1 | F. exact | 1.000 |
| *Chelostoma florisomne* | 1.0 | 1.0 | - | - | 1 | 1 | F. exact | 1.000 |
| *Chelostoma rapunculi* | 1.0 ± 0.0 | - | - | - | 3 | 0 | F. exact | 0.262 |
| *Coelioxys elongata* | 2.0 | - | - | - | 3 | 0 | F. exact | 0.262 |
| *Colletes daviesanus* | 1.0 | 2.0 | W = 0 | 1.000 | 1 | 1 | F. exact | 1.000 |
| *Colletes fodiens* | 1.5 ± 0.7 | - | - | - | 2 | 0 | F. exact | 0.504 |
| *Colletes similis* | 1.0 ± 0.0 | - | - | - | 2 | 0 | F. exact | 0.504 |
| *Dasypoda altercator* | - | 1.0 | - | - | 0 | 1 | F. exact | 0.444 |
| *Epeolus cruciger* | 1.0 ± 0.0 | - | - | - | 2 | 0 | F. exact | 0.504 |
| *Eucera longicornis* | 1.0 ± 0.0 | 1.0 | - | - | 2 | 1 | F. exact | 1.000 |
| *Evylaeus albipes* | - | 1.0 | - | - | 0 | 1 | F. exact | 0.444 |
| *Evylaeus calceatus* | 1.2 ± 0.4 | 1.5 ± 0.7 | W = 8 | 0.508 | 6 | 2 | F. exact | 0.450 |
| *Evylaeus fulvicornis* | 1.3 ± 0.6 | 1.0 ± 0.0 | W = 3 | 0.505 | 3 | 3 | F. exact | 1.000 |
| *Evylaeus laticeps* | 2.0 ± 1.4 | - | - | - | 12 | 0 | F. exact | **0.005** |
| *Evylaeus leucopus* | - | 1.0 | - | - | 0 | 1 | F. exact | 0.444 |
| *Evylaeus malachurus* | - | 1.0 | - | - | 0 | 1 | F. exact | 0.444 |
| *Evylaeus morio* | 1.3 ± 0.5 | 1.5 ± 0.7 | W = 13.5 | 0.622 | 11 | 2 | F. exact | 0.103 |
| *Evylaeus nitidiusculus* | 1.0 ± 0.0 | 1.0 ± 0.0 | - | - | 2 | 2 | F. exact | 1.000 |
| *Evylaeus nitidulus* | 1.0 ± 0.0 | - | - | - | 2 | 0 | F. exact | 0.504 |
| *Evylaeus pauxillus* | 1.6 ± 1.1 | 2.2 ± 1.6 | W = 26 | 0.463 | 7 | 6 | F. exact | 1.000 |
| *Evylaeus politus* | 3.2 ± 2.9 | 2.3 ± 1.5 | W = 18 | 0.889 | 13 | 3 | F. exact | 0.130 |
| *Evylaeus tricinctus* | 1.0 | - | - | - | 1 | 0 | F. exact | 1.000 |
| *Evylaeus villosulus* | - | 1.4 ± 0.9 | - | - | 0 | 5 | F. exact | **0.022** |
| *Halictus compressus* | - | 1.0 ± 0.0 | - | - | 0 | 3 | F. exact | 0.095 |
| *Halictus laevigatum* | 1.0 | 1.3 ± 0.6 | W = 2 | 1.000 | 1 | 3 | F. exact | 0.320 |
| *Halictus maculatus* | 2.0 ± 1.5 | 1.0 ± 0.0 | W = 1.5 | 0.414 | 2 | 3 | F. exact | 0.646 |
| *Halictus quadricinctus* | 1.0 | - | - | - | 1 | 0 | F. exact | 1.000 |
| *Halictus rubicundus* | 1.0 | - | - | - | 1 | 0 | F. exact | 1.000 |
| *Halictus sexcinctus* | 1.0 | - | - | - | 3 | 0 | F. exact | 0.262 |
| *Heriades truncorum* | 1.0 | - | - | - | 1 | 0 | F. exact | 1.000 |
| *Hoplitis adunca* | 1.0 | - | - | - | 1 | 0 | F. exact | 1.000 |
| *Hoplitis leucomelana* | 1.0 | 1.0 | - | - | 1 | 1 | F. exact | 1.000 |
| *Hoplitis spinulosa* | 1.0 | - | - | - | 1 | 0 | F. exact | 1.000 |
| *Hylaeus annularis* | 1.0 ± 0.0 | 1.0 | - | - | 2 | 1 | F. exact | 1.000 |
| *Hylaeus brevicornis* | 2.5 ± 0.6 | - | - | - | 4 | 0 | F. exact | 0.142 |
| *Hylaeus cardioscapus* | 1.0 | - | - | - | 1 | 0 | F. exact | 1.000 |
| *Hylaeus communis* | 2.0 ± 1.15 | - | - | - | 4 | 0 | F. exact | 0.142 |
| *Hylaeus confusus* | 1.0 ± 0.0 | - | - | - | 3 | 0 | F. exact | 0.262 |
| *Hylaeus cornutus* | 1.0 | 1.0 | - | - | 1 | 1 | F. exact | 1.000 |
| *Hylaeus difformis* | 1.0 | - | - | - | 1 | 0 | F. exact | 1.000 |
| *Hylaeus gredleri* | - | 2.0 | - | - | 0 | 1 | F. exact | 0.444 |
| *Hylaeus hyalinatus* | 1.3 ± 0.6 | - | - | - | 3 | 0 | F. exact | 0.262 |
| *Hylaeus nigritus* | 1.0 | - | - | - | 1 | 0 | F. exact | 1.000 |
| *Hylaeus pictipes* | 1.0 ± 0.0 | - | - | - | 2 | 0 | F. exact | 0.504 |
| *Hylaeus signatus* | 2.3 ± 1.8 | - | - | - | 6 | 0 | F. exact | 0.071 |
| *Lasioglossum lativentre* | 1.0 ± 0.0 | 1.0 | - | - | 2 | 1 | F. exact | 1.000 |
| *Lasioglossum leucozonium* | - | 1.7 ± 1.2 | - | - | 0 | 3 | F. exact | 0.095 |
| *Lasioglossum majus* | 1.0 | - | - | - | 1 | 0 | F. exact | 1.000 |
| *Lasioglossum sexnotatum* | 1.0 ± 0.0 | 1.0 ± 0.0 | - | - | 3 | 2 | F. exact | 1.000 |
| *Lasioglossum xanthopus* | 1.2 ± 0.4 | - | - | - | 5 | 0 | F. exact | 0.142 |
| *Lasioglossum zonulum* | - | 1.0 | - | - | 0 | 1 | F. exact | 0.444 |
| *Megachile versicolor* | 1.0 ± 0.0 | - | - | - | 2 | 0 | F. exact | 0.504 |
| *Melitta leporina* | 1.0 | - | - | - | 1 | 0 | F. exact | 1.000 |
| *Melitta nigricans* | - | 1.0 | - | - | 0 | 1 | F. exact | 0.444 |
| *Nomada distinguenda* | - | 1.0 | - | - | 0 | 1 | F. exact | 0.444 |
| *Nomada flavoguttata* | 1.0 | - | - | - | 1 | 0 | F. exact | 1.000 |
| *Osmia aurulenta* | 1.0 | 1.0 | - | - | 1 | 1 | F. exact | 1.000 |
| *Panurgus calcaratus* | - | 1.0 ± 0.0 | - | - | 0 | 2 | F. exact | 0.203 |
| *Rhophitoides canus* | 1.8 ± 1.0 | - | - | - | 4 | 0 | F. exact | 0.142 |
| *Seladonia confusa* | 1.0 ± 0.0 | - | - | - | 2 | 0 | F. exact | 0.504 |
| *Seladonia subaurata* | 2.6 ± 1.6 | 1.5 ± 0.8 | W = 20 | 0.127 | 12 | 6 | F. exact | 0.574 |
| *Seladonia tumulorum* | 1.7 ± 1.3 | 1.5 ± 1.0 | W = 20 | 0.815 | 11 | 4 | F. exact | 0.362 |
| *Sphecodes crassus* | 1.0 ± 0.0 | - | - | - | 2 | 0 | F. exact | 0.504 |
| *Sphecodes ephippius* | 1.5 ± 0.7 | - | - | - | 2 | 0 | F. exact | 0.504 |
| *Sphecodes ferruginatus* | 1.0 | - | - | - | 1 | 0 | F. exact | 1.000 |
| *Sphecodes longulus* | 1.0 | - | - | - | 1 | 0 | F. exact | 1.000 |
| *Sphecodes punctipes* | 1.0 | - | - | - | 1 | 0 | F. exact | 1.000 |
| *Sphecodes reticulatus* | - | 1.0 | - | - | 0 | 1 | F. exact | 0.444 |

b)

| Species | Abundance | | | | Occurrence | | | |
| --- | --- | --- | --- | --- | --- | --- | --- | --- |
|  | Embankment | Grassland | Statistic | P | Embankment | Grassland | Statistic | P |
| *Aglais urticae* | 1.8 ± 0.9 | 1.5 ± 0.6 | W =21 | 0.576 | 13 | 4 | F. exact | 0.239 |
| *Anthocharis cardamines* | 1.1 ± 0.3 | 1.3 ± 0.5 | W = 37 | 0.435 | 10 | 7 | F. exact | 1.000 |
| *Apatura ilia* | 1.0 | 1.0 | - | - | 1 | 1 | F. exact | 1.000 |
| *Apatura iris* | 1.0 | - | - | - | 1 | 0 | F. exact | 1.000 |
| *Aphanthopus hyperanthus* | 2.4 ± 1.6 | 2.8 ± 1.7 | W = 180 | 0.363 | 18 | 17 | $\chi^{2}=0.2$ | 0.633 |
| *Araschnia levana* | 2.4 ± 1.4 | 1.9 ± 1.1 | W = 76 | 0.460 | 14 | 13 | $\chi^{2}=0.2$ | 0.683 |
| *Argynnis adippe* | 1.0 | - | - | - | 1 | 0 | F. exact | 1.000 |
| *Argynnis aglaja* | 1.0 ± 0.0 | 1.0 ± 0.0 | - | - | 2 | 2 | F. exact | 1.000 |
| *Argynnis paphia* | 1.0 ± 0.0 | 1.0 | - | - | 2 | 1 | F. exact | 1.000 |
| *Aricia agestis* | 2.4 ± 1.3 | 1.2 ±0.5 | W = 7 | 0.086 | 9 | 4 | F. exact | 0.529 |
| *Boloria dia* | 2.1 ± 1.3 | 2.2 ± 1.1 | W = 49 | 0.620 | 17 | 5 | F. exact | 0.174 |
| *Boloria selene* | - | 1.5 ± 0.7 | - | - | 0 | 2 | F. exact | 0.203 |
| *Brenthis ino* | 1.0 | 2.0 ± 1.0 | - | - | 1 | 3 | F. exact | 0.320 |
| *Callophrys rubi* | 1.8 ± 1.2 | 1.0 ± 0.0 | W = 14 | 0.124 | 13 | 4 | F. exact | 0.239 |
| *Carcharodus alceae* | 1.7 ±1.2 | - | - | - | 6 | 0 | F. exact | 0.071 |
| *Celastrina argiolus* | 1.2 ± 0.4 | 1.5 ± 0.7 | W = 8 | 0.508 | 6 | 2 | F. exact | 0.450 |
| *Coenonympha arcania* | 1.5 ± 0.7 | 1.0 | - | - | 13 | 1 | F. exact | **0.021** |
| *Coenonympha glycerion* | 2.2 ± 1.6 | 1.8 ± 0.75 | W = 15 | 1.000 | 6 | 5 | F. exact | 1.000 |
| *Coenonympha pamphilus* | 2.8 ± 1.9 | 2.6 ± 2.1 | W = 144 | 0.334 | 22 | 16 | $\chi^{2}=0.0$ | 0.922 |
| *Colias croceus* | 1.6 ± 0.8 | - | - | - | 7 | 0 | F. exact | **0.037** |
| *Colias hyale* | 1.7 ± 0.8 | 1.2 ± 0.4 | W = 12 | 0.159 | 7 | 6 | F. exact | 1.000 |
| *Cupido argiades* | 1.4 ± 0.5 | 1.0 | - | - | 11 | 1 | F. exact | **0.039** |
| *Cupido minimus* | 2.1 ± 1.0 | - | - | - | 9 | 0 | F. exact | **0.019** |
| *Erynnis tages* | 1.7 ± 0.7 | 1.8 ± 0.9 | W = 56 | 1.000 | 14 | 8 | F. exact | 0.791 |
| *Gonepteryx rhamni* | 2.7 ± 1.9 | 2.4 ± 1.7 | W = 73 | 0.930 | 15 | 10 | $\chi^{2}=0.1$ | 0.797 |
| *Hesperia comma* | 1.5 ± 0.7 | 1.0 ± 0.0 | W = 1.5 | 0.414 | 2 | 3 | F. exact | 0.646 |
| *Hyponephele lycaon* | 1.7 ± 0.6 | - | - | - | 3 | 0 | F. exact | 0.262 |
| *Inachis io* | 2.6 ±1.8 | 1.6 ± 0.7 | W = 52 | 0.081 | 14 | 12 | $\chi^{2}=0.1$ | 0.809 |
| *Issoria lathonia* | 2.3 ± 1.7 | 1.4 ± 0.7 | W = 56 | 0.058 | 19 | 10 | $\chi^{2}=0.6$ | 0.457 |
| *Lasiommata maera* | 2.7 ± 1.7 | 1.1 ± 0.4 | W = 19.5 | **0.006** | 18 | 7 | F. exact | 0.302 |
| *Leptidea reali* | 1.0 ± 0.0 | 1.0 | - | - | 2 | 1 | F. exact | 1.000 |
| *Lycaena alciphron* | 2.5 ± 0.7 | - | - | - | 2 | 0 | F. exact | 0.504 |
| *Lycaena dispar* | 2.0 | 1.0 ± 0.0 | - | - | 1 | 5 | F. exact | 0.092 |
| *Lycaena hippothoe* | - | 1.5 ± 0.7 | - | - | 0 | 2 | F. exact | 0.203 |
| *Lycaena phlaeas* | 2.2 ± 2.3 | 1.1 ± 0.4 | W = 38 | 0.166 | 16 | 7 | F. exact | 0.429 |
| *Lycaena tityrus* | 1.94 ± 1.0 | 1.0 ± 0.0 | W = 14 | **0.046** | 18 | 4 | F. exact | 0.057 |
| *Lycaena virgaureae* | 2.3 ± 1.6 | 1.5 ± 0.7 | W = 10.5 | 0.615 | 14 | 2 | F. exact | **0.034** |
| *Maculinea nausithous* | 1.0 ± 0.0 | 2.0 ± 1.2 | W = 6 | 0.402 | 2 | 4 | F. exact | 0.395 |
| *Maculinea teleius* | - | 5.0 ± 4.2 | - | - | 0 | 2 | F. exact | 0.203 |
| *Maniola jurtina* | 2.8 ± 1.2 | 2.7 ± 2.3 | W = 125 | 0.255 | 23 | 14 | $\chi^{2}=0.2$ | 0.626 |
| *Melanargia galathea* | 2.9 ± 1.8 | 1.8 ± 1.3 | W = 26.5 | 0.130 | 19 | 5 | F. exact | 0.110 |
| *Melitaea athalia* | 1.0 | 1.0 | - | - | 1 | 1 | F. exact | 1.000 |
| *Nymphalis antiopa* | 1.0 ± 0.0 | 1.0 | - | - | 2 | 1 | F. exact | 1.000 |
| *Nymphalis polychloros* | 1.4 ± 0.5 | 1.0 | - | - | 12 | 1 | F. exact | **0.022** |
| *Ochlodes sylvanus* | 1.9 ± 0.6 | 1.2 ± 0.4 | W = 10 | **0.030** | 9 | 6 | F. exact | 1.000 |
| *Papilio machaon* | 1.4 ± 0.5 | 1.0 ± 0.0 | W = 12.5 | 0.106 | 9 | 5 | F. exact | 0.759 |
| *Parargae aegeria* | - | 1.5 ± 0.7 | - | - | 0 | 2 | F. exact | 0.203 |
| *Pieris brassicae* | 3.2 ± 1.4 | 2.7 ± 1.7 | W = 101 | 0.242 | 19 | 4 | F. exact | 0.057 |
| *Pieris napi* | 1.2 ± 0.5 | 2.2 ± 1.8 | W = 12.5 | 0.560 | 4 | 5 | F. exact | 0.715 |
| *Pieris rapae* | 5.8 ± 4.0 | 4.3 ± 2.9 | W = 150 | 0.248 | 24 | 16 | $\chi^{2}=0.1$ | 0.768 |
| *Plebejus argus* | 2.7 ± 1.6 | 1.0 | - | - | 9 | 1 | F. exact | 0.072 |
| *Plebejus argyrognomon* | 1.8 ± 1.0 | 1.0 | - | - | 11 | 1 | F. exact | **0.039** |
| *Polygonia c-album* | 4.6 ± 3.6 | 4.1 ± 2.8 | W = 103.5 | 0.803 | 20 | 11 | $\chi^{2}=0.4$ | 0.503 |
| *Polyommatus bellargus* | 3.0 | - | - | - | 1 | 0 | F. exact | 1.000 |
| *Polyommatus coridon* | 2.8 ± 1.5 | - | - | - | 11 | 0 | F. exact | **0.010** |
| *Polyommatus icarus* | 4.4 ± 2.8 | 2.4 ± 1.8 | W = 58 | **0.044** | 19 | 11 | $\chi^{2}=0.3$ | 0.575 |
| *Polyommatus semiargus* | 1.3 ± 0.6 | 1.0 ± 0.0 | W = 3 | 0.505 | 3 | 3 | F. exact | 1.000 |
| *Pontia edusa* | 1.2 ± 0.4 | 1.0 ± 0.0 | W = 10 | 0.540 | 6 | 4 | F. exact | 1.000 |
| *Pyrgus alveus* | 1.7 ± 0.6 | - | - | - | 3 | 0 | F. exact | 0.262 |
| *Pyrgus malvae* | 2.0 ± 0.9 | 1.6 ± 0.6 | W = 25 | 0.451 | 13 | 5 | F. exact | 0.390 |
| *Satyrium ilicis* | 1.0 | - | - | - | 1 | 0 | F. exact | 1.000 |
| *Thecla betulae* | 2.0 ± 1.4 | - | - | - | 2 | 0 | F. exact | 0.504 |
| *Thymelicus acteon* | 2.2 ±0.8 | - | - | - | 6 | 0 | F. exact | 0.071 |
| *Thymelicus lineola* | 2.8 ±1.6 | 2.6 ±1.4 | W = 116 | 0.887 | 15 | 16 | $\chi^{2}=0.5$ | 0.471 |
| *Thymelicus silvestris* | 2.4 ± 1.7 | 2.0 ± 1.2 | W = 24 | 0.718 | 11 | 5 | F. exact | 0.554 |
| *Vanessa atalanta* | 2.2 ± 1.7 | 1.6 ± 0.7 | W = 45.5 | 0.368 | 13 | 9 | F. exact | 1.000 |

c)

| Species | Abundance | | | | Occurrence | | | |
| --- | --- | --- | --- | --- | --- | --- | --- | --- |
|  | Embankment | Grassland | Statistic | P | Embankment | Grassland | Statistic | P |
| *Anasimya lineata* | 1.0 | - | - | - | 1 | 0 | F. exact | 1.000 |
| *Cheilosia urbana* | 4.0 | - | - | - | 1 | 0 | F. exact | 1.000 |
| *Cheilosia vernalis* | 1.0 | - | - | - | 1 | 0 | F. exact | 1.000 |
| *Chrysotoxum cautum* | 2.0 | 1.0 | - | - | 1 | 1 | F. exact | 1.000 |
| *Chrysotoxum festivum* | - | 1.0 | - | - | 0 | 1 | F. exact | 0.444 |
| *Chrysotoxum lineare* | - | 1.0 | - | - | 0 | 1 | F. exact | 0.444 |
| *Dasysyrphus venustus* | 2.0 | - | - | - | 1 | 0 | F. exact | 1.000 |
| *Epistrophe flava* | 1.0 | - | - | - | 1 | 0 | F. exact | 1.000 |
| *Epistrophe nitidicollis* | 1.0 | - | - | - | 1 | 0 | F. exact | 1.000 |
| *Episyrphus balteatus* | 1.0 ± 0.0 | 2 .0± 1.2 | W = 12 | 0.168 | 3 | 5 | F. exact | 0.447 |
| *Eristalinus aeneus* | 2.0 ± 1.0 | 1.0 ± 0.0 | W = 1.5 | 0.197 | 3 | 3 | F. exact | 1.000 |
| *Eristalinus sepulchralis* | 1.0 ± 0.0 | - | - | - | 3 | 0 | F. exact | 0.262 |
| *Eristalis arbustorum* | 4.2 ± 3.1 | 1.8 ±1.1 | W = 58.5 | **0.002** | 19 | 16 | $\chi^{2}=0.0$ | 0.822 |
| *Eristalis horticola* | - | 1.0 | - | - | 0 | 1 | F. exact | 0.444 |
| *Eristalis intricaria* | 1.0 | - | - | - | 1 | 0 | F. exact | 1.000 |
| *Eristalis nemorum* | 1.0 | - | - | - | 1 | 0 | F. exact | 1.000 |
| *Eristalis pertinax* | 1.0 ± 0.0 | - | - | - | 2 | 0 | F. exact | 0.504 |
| *Eristalis similis* | 1.0 | 1.0 | - | - | 1 | 1 | F. exact | 1.000 |
| *Eristalis tenax* | 1.7 ± 0.8 | 1.6 ± 0.6 | W = 30 | 1.000 | 12 | 5 | F. exact | 0.391 |
| *Eumerus strigatus* | - | 1.0 | - | - | 0 | 1 | F. exact | 0.444 |
| *Eupeodes corollae* | 1.0 | - | - | - | 1 | 0 | F. exact | 1.000 |
| *Helophilus hybridus* | - | 1.0 | - | - | 0 | 1 | F. exact | 0.444 |
| *Helophilus pendulus* | 1.0 | - | - | - | 1 | 0 | F. exact | 1.000 |
| *Helophilus trivittatus* | 1.0 ± 0.0 | 1.2 ±0.4 | W = 10.5 | 0.637 | 3 | 6 | F. exact | 0.278 |
| *Melanostoma mellinum* | 1.9 ± 1.4 | 1.3 ± 0.5 | W = 37.5 | 0.506 | 10 | 9 | F. exact | 0.788 |
| *Melanostoma scalare* | - | 1.0 ± 0.0 | - | - | 0 | 3 | F. exact | 0.095 |
| *Myathropa florea* | 1.0 ± 0.0 | - | - | - | 2 | 0 | F. exact | 0.504 |
| *Neoascia meticulosa* | 1.0 | - | - | - | 1 | 0 | F. exact | 1.000 |
| *Neoascia podgarica* | 2.0 | - | - | - | 1 | 0 | F. exact | 1.000 |
| *Pipiza festiva* | - | 1.0 | - | - | 0 | 1 | F. exact | 0.444 |
| *Pipizella viduata* | 1.8 ± 0.8 | 1.2 ±0.4 | W = 8.5 | 0.100 | 6 | 6 | F. exact | 0.750 |
| *Platycheirus angustatus* | 1.0 | - | - | - | 1 | 0 | F. exact | 1.000 |
| *Platycheirus clypeatus* | 1.0 | 1.0 | - | - | 1 | 1 | F. exact | 1.000 |
| *Platycheirus europaeus* | 1.0 ± 0.0 | - | - | - | 2 | 0 | F. exact | 0.504 |
| *Platycheirus fulviventris* | 1.0 | - | - | - | 1 | 0 | F. exact | 1.000 |
| *Platycheirus peltatus* | 1.0 ± 0.0 | 1.0 | - | - | 2 | 1 | F. exact | 1.000 |
| *Platycheirus scutatus* | 1.0 | 1.0 | - | - | 1 | 1 | F. exact | 1.000 |
| *Scaeva pyrastri* | 1.0 ± 0.0 | - | - | - | 2 | 0 | F. exact | 0.504 |
| *Sphaerophoria batava* | 1.0 ± 0.0 | 1.0 | - | - | 2 | 1 | F. exact | 1.000 |
| *Sphaerophoria rueppelli* | 1.0 | 2.0 ± 1.4 | - | - | 1 | 4 | F. exact | 0.173 |
| *Sphaerophoria scripta* | 6.3 ± 5.4 | 7.6 ±4.2 | W = 240 | 0.226 | 23 | 17 | $\chi^{2}=0.0$ | 0.950 |
| *Sphaerophoria taeniata* | - | 1.0 ± 0.0 | - | - | 0 | 2 | F. exact | 0.203 |
| *Syritta pipiens* | 2.4 ± 2.0 | 3.4 ±3.5 | W = 122.5 | 0.165 | 17 | 12 | $\chi^{2}=0.0$ | 0.879 |
| *Triglyphus primus* | - | 1.0 | - | - | 0 | 1 | F. exact | 0.444 |
| *Volucella bombylans* | - | 1.5 ± 0.7 | - | - | 0 | 2 | F. exact | 0.203 |
| *Xanthogramma pedissequum* | 1.3 ± 0.6 | - | - | - | 3 | 0 | F. exact | 0.262 |
